# On the conundrum of cognitive impairment due to depressive disorder in older patients

**DOI:** 10.1371/journal.pone.0231111

**Published:** 2020-04-02

**Authors:** Claudia E. Lanza, Karolina Sejunaite, Charlotte Steindel, Ingo Scholz, Matthias W. Riepe

**Affiliations:** Department of Psychiatry and Psychotherapy II, Mental Health & Old Age Psychiatry, Ulm University, Ulm, Germany; Nathan S Kline Institute, UNITED STATES

## Abstract

**Objectives:**

Depressive symptoms and cognitive impairment often concur in older persons. Differentiating the cause of cognitive impairment in older persons with Depressive Disorder (DD) from other diseases such as Alzheimer’s Disease (AD) is challenging. The goal of this study was to characterize cognitive impairment in older persons with DD.

**Design:**

Cross-sectional retrospective observational clinical cohort study using patient records from 2014 to 2018.

**Setting:**

Gerontopsychiatric services of Ulm University at Bezirkskrankenhaus Günzburg serving as primary psychiatric care institution and tertiary referral center for psychiatric care for older persons.

**Partcipants:**

DD was diagnosed according to ICD-10 criteria. When indicated by the medical history or neuropsychological assessment further diagnostic procedures were initiated. Cerebrospinal fluid (CSF) tap was routinely the first additional procedure. If patients did not consent to CSF tap or contraindications were present, ^18^F-fluordesoxyglucose-PET (FDG-PET) or Amyloid-PET (Am-PET) were performed.

**Materials and methods:**

Extensive neuropsychological test battery to assess cognitive profile.

**Results:**

457 subjects were diagnosed with DD (DD-all; age 50–94; 159 males, 298 females). Biomarkers were assessed in 176 persons; in 90 of these subjects AD-biomarkers were negative (DD-BM-; age 54–89; 40 males, 50 females), and in 86 subjects at least one biomarker was compatible with AD (DD-BM+; age 60–90; 31 males, 55 females). Cognitive performance was below healthy controls (HC; n = 56; age 50–80; 30 males, 26 females) for all groups of patients with DD. With case-control matching of HC and DD-BM- we find that executive functions are impaired in about one out of three and delayed recall in about two out of three patients with DD.

**Conclusion:**

Cognitive impairment is frequent in older persons with DD. Cognitive profile in older patients with DD without and with biomarkers of AD is not distinguishable. Therefore, cognitive impairment due to DD should be diagnosed after exclusion of comorbid AD.

## Introduction

Cognitive deficits are frequent in patients with Depressive Disorder (DD) [1]. The majority of previous studies investigated younger patients. It is generally thought that symptoms of DD are alike across adulthood [2, 3]. However, due to a lack of studies including older persons it remains unclear whether the extent of the association of DD with cognitive impairment is independent of age.

Until now cognitive symptoms are not included in the diagnostic classifications of ICD-10 or the Diagnostic and Statistical Manual of Mental Disorders (DSM-5) with the same elaborateness as other symptoms of DD. Therefore, hitherto existing literature reports on the frequency and pattern of cognitive impairment in patients with DD are subject to selection bias because patients with cognitive impairment may have been excluded. Likewise, assessment bias cannot be ruled out because cognition may not have been assessed with sensitive methods. Lastly, reporting bias may be present because information on cognitive performance of patients with DD is undervalued and not reported.

Organic diseases of the central nervous system are rare in younger persons. Thus, characterization of the pattern of cognitive deficits is sufficient to delineate the profile of cognitive impairment due to DD in younger subjects. Studies in older persons often assume the same procedure and diagnose the cause of cognitive impairment on clinical and neuropsychological grounds [4–6] despite other studies reporting that distinction between cognitive impairment due to DD and cognitive impairment due to AD is difficult if not impossible on clinical and neuropsychological grounds [7, 8].

In contrast to younger persons, older adults with DD have several co-morbidities affecting cognitive performance [3] of which Alzheimer’s disease (AD) is the most common. The proportion of patients with both depressive symptoms and cognitive impairment is going to increase in the next two decades [9]. On grounds of current clinical practice, the etiological cause of cognitive impairment in older persons cannot be diagnosed reliably. This lack of knowledge impedes not only the diagnosis of DD in older persons but also an appropriate treatment.

The course of DD on treatment has been reported to be worse in older subjects than in younger subjects although no association has been found with established risk factors for worse treatment outcome [10]. It has been speculated that the worse outcome of treatment may be related to cognitive impairment [10] but it remains unclear whether this addressed cognitive impairment due to DD or cognitive impairment due to unknown co-morbidities.

This conundrum is even more complicated since cognitive impairment may occur separately from episodes of low mood [1]. Patients’ complaints about deteriorating cognitive function often results from depressive symptoms and complaints about depression often result from impaired cognition [11, 12]. It has been observed that many older individuals who are cognitively impaired during a depressive episode remain cognitively impaired on remission of depression [13]. This raises the question whether diseases other than DD may have contributed to cognitive impairment in some of these patients [14]. Likewise, a substantial proportion of older depressed individuals, who are cognitively intact when depressed, have been found to be cognitively impaired on follow-up after one year [13]. Again, this raises doubts on whether some of these subjects already had had concomitant diseases such as preclinical AD in the first place [15, 16]. In line with this argument the higher risk of dementia over the course of DD has been interpreted as depression being a prodrome of dementia rather than a predictor [17]. Even longitudinal observation of older patients with DD over more than 10 years [17] is not sufficient to resolve the conundrum of cognitive impairment in older subjects with DD. The reason for this insufficiency is that amyloid pathology precedes clinical symptoms of dementia up to several decades [18]. Thus, diagnosis of a depressive symptom years ahead of diagnosing cognitive impairment as the lead symptom may be confounded by amyloid pathology already being present at the time of diagnosing DD.

Disentangling the intricate issues of cognition and depression on clinical and neuropsychological grounds is impossible. It cannot be resolved with clinical and neuropsychological methods whether a given cognitive symptom is due to DD or whether DD itself is a consequence of a comorbid condition known to foster cognitive symptoms in older persons. Nevertheless, sophisticated neuropsychological assessment is necessary to objectify cognitive impairment reported by patients. In addition, the most common cause of cognitive impairment in older persons, AD, needs to be ruled out with other methods.

A recent consensus paper came to the conclusion that AD is ruled out when CSF-biomarkers of AD are negative [19]. Similarly it was argued from longitudinal data that in the sequence of events a decrease of Abeta1,42 comes prior to cognitive impairment [20–22]. Thus, normal levels of Abeta1,42 argue in favor of cognitive symptoms not being due to underlying AD. Demonstrating normal levels of tau-protein supports this and helps to distinguish subjects with age-associated neurodegenerative diseases or even vascular disease [23–25].

The goal of the present study was to tackle the conundrum of cognitive impairment due to DD by analyzing patients with DD in whom comorbid AD has been excluded with use of AD-biomarkers (amyloid- and tau-proteins in cerebrospinal fluid, ^18^F-fluordesoxyglucose-PET (FDG-PET), or amyloid-PET (Am-PET)). We hypothesized that cognitive impairment is frequent in older persons with DD even if AD as the most common cause for cognitive impairment in older age is ruled out.

## Materials and methods

We performed an observational clinical cohort study and used patient records of the gerontopsychiatric services at Ulm University at Bezirkskrankenhaus Günzburg from 2014 to 2018. The study received approval of the ethics committee of Ulm University (289/18) and was conducted according to the ethical standards of Ulm University and the guidelines outlined in the declaration of Helsinki.

### Participants

**Fig 1:**
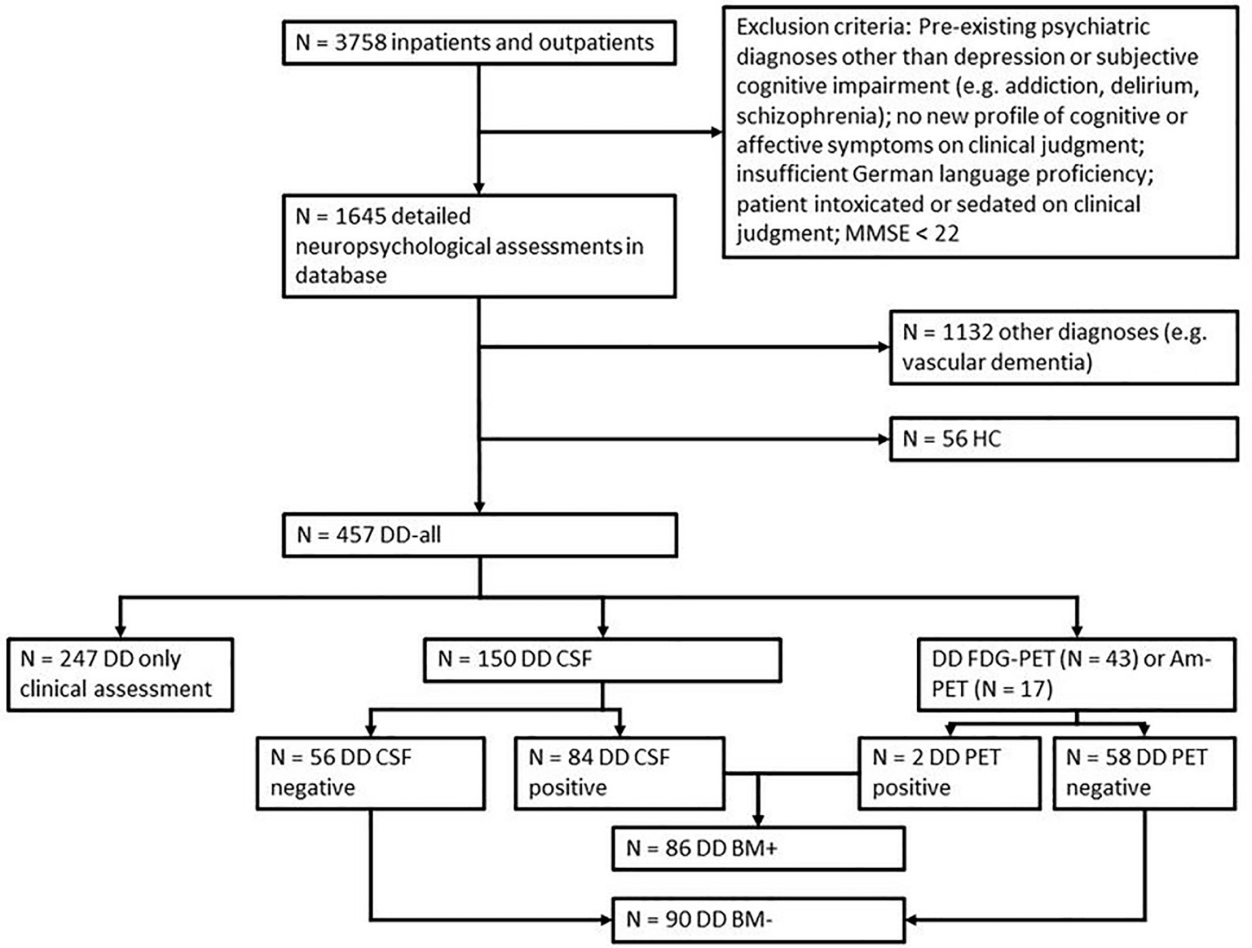
Flow-chart of patient selection procedures. Regardless of complaint or referral diagnosis it is our standard practice to assess patients with complaints of both depressed mood and impaired cognition with a detailed neuropsychological assessment if none of the exclusion criteria is fulfilled. DD: Depressive Disorder; HC: Healthy controls; BM: Biomarker; CSF: Cerebrospinal fluid; FDG-PET: ^18^F-fluordesoxyglucose-PET; Am-PET: Amyloid-PET; PET: positron emission tomography; MMSE: Mini-Mental Status Examination.

Gerontopsychiatric services of Ulm University at Bezirkskrankenhaus Günzburg serve as both a primary gerontopsychiatric service for a rural catchment area of about 650.000 people and a University affiliated tertiary referral center for gerontopsychiatry. As such patients are referred to this unit by general practitioners, neurologists and psychiatrists in private practice, as well as surrounding hospitals. Reflecting this practice and clinical reality these patients often complain about both mood change and memory impairment and many patients lack sufficient introspection to give a clear picture of their primary complaint, which not always accords with the referral diagnosis and report by proxies. Regardless of complaint or referral diagnosis it is our standard practice to assess patients with complaints of both depressed mood and impaired cognition with a detailed neuropsychological assessment if none of the exclusion criteria is fulfilled (Fig 1). We included all inpatients and outpatients from 2014 to 2018.

Persons were diagnosed as healthy controls (HC) upon unremarkable Medical History without cognitive decline compared to previous capabilities in everyday life and normal performance on neuropsychological assessment. For ethical reasons and considering normality of findings no further diagnostic procedures were initiated. Table 1 shows the demographic variables and performance of healthy controls (HC).

**Table 1:** Demographic variables.

	HC				DD-all					
N	56				457					
male/female	30 / 26				159 / 298					
education basic / intermedium / high / missing	18 / 15 / 23 / 0				308 / 90 / 48 / 11					
	Median	Min	Max	N	Median	Min	Max	N	Mann-Whitney-U-Test HC vs. DD-all asymptotic significance	z-value
**Age (mean/SD)**	66.5	50	80	56	74,0	50,0	94,0	457	0.000	-4.394
**MMSE (mean/SD)**	30	28	30	56	28,0	16,0	30,0	443	0.000	7.476
**GDS (mean/SD)**	1	0	8	50	7,0	0,0	15,0	423	0.000	-8.677
**Digit Span forward**	8	4	12	56	7,0	1,0	12,0	442	0.000	4.399
**Digit Span backward**	6	2	11	56	4,0	0,0	12,0	442	0.000	5.698
**Block Span forward**	7	4	10	56	6,0	1,0	12,0	429	0.000	3.489
**Block Span backward**	6	3	10	56	5,0	0,0	10,0	429	0.000	5.771
**CVLT1**	5	0	10	56	4,0	0,0	9,0	444	0.000	4.283
**CVLT2**	8	4	13	56	5.5	0,0	13,0	444	0.000	6.088
**CVLT3**	10	4	14	56	7,0	2,0	16,0	442	0.000	6.781
**CVLT4**	11	4	16	56	8,0	1,0	16,0	440	0.000	6.429
**CVLT5**	12.5	1	16	56	8,0	1,0	16,0	440	0.000	7.172
**CVLT total recall**	43	21	64	56	32,0	8,0	66,0	440	0.000	7.178
**CVLT del. free recall**	11	2	16	56	6,0	0,0	16,0	435	0.000	7.348
**CVLT del. cued recall**	12.5	5	16	56	8,0	0,0	16,0	436	0.000	7.081
**CVLT recognition**	16	13	16	56	15,0	0,0	16,0	433	0.000	4.516
**CVLT false positve**	0	0	4	56	1,0	0,0	21,0	431	0.000	-5.326
**Clock drawing**	1	1	4	56	2,0	1,0	5,0	431	0.000	-5.031
**TMT-A**	38	13	180	56	62,0	10,0	277,0	428	0.000	-6.095
**TMT-B**	95	28	270	56	156,0	28,0	345,0	371	0.000	-6.764
**Semantic Fluency**	21.5	12	38	56	16,0	3,0	37,0	435	0.000	5.887
**Phonematic Fluency P**	9	1	18	56	6,0	0,0	19,0	432	0.000	5.613
**Phonematic Fluency S**	13	2	22	53	9,0	0,0	23,0	422	0.000	5.296

HC: Healthy controls; DD-all: all patients with DD; CVLT: California Verbal Learning Test; GDS: 15-item Geriatric Depression Scale; MMSE: Mini-Mental-Status-Examination; Semantic fluency: category animals; TMT-A and TMT-B: Trail-Making-Test A and B

In younger patients with DD, organic diseases of the central nervous system are rarely present, and it is usually possible to determine how many episodes have occurred previously. Similar to an approach reported in the literature [26] we diagnosed the depressive mood phenotypically according to ICD-10 criteria for depressive disorder without either organic exclusions or diagnostic hierarchy rules.

Further diagnostic procedures in persons with DD were initiated when on clinical judgment the report on cognitive decline comprised a slowly progressing course of impairment over many months, and when at least a moderate decline of episodic memory was found in the neuropsychological assessment, or when patients remained worried even after clinical and neuropsychological examination and requested further diagnostic measures. The indeterminateness of the decision-making algorithm reflects the hitherto existing vague knowledge on the pattern of cognitive impairment due to DD and due to AD. We performed a cerebrospinal fluid (CSF) tap to rule out organic causes of cognitive impairment. If patients did not consent to CSF tap or contraindications were present (e.g. anticoagulation), FDG-PET or Am-PET was performed. We initiated an additional FDG-PET or Am-PET in some patients when clinical judgment and results of CSF tap differed.

### Assessments

#### Geriatric Depression Scale (GDS)

The short version of the Geriatric Depression scale [27] is a 15-item questionnaire to designed to assess symptoms of depression in older adults. Participants are asked to answer each item with yes or no. Scores above 4 are compatible with depressive syndrome (5–8 indicate mild depression; 9–11 indicate moderate depression; and 12–15 indicate severe depression).

#### Mini-Mental Status Examination (MMSE)

The MMSE is a commonly used screening instrument to detect cognitive impairment as well as stage the severity of dementia by assessing a range of cognitive functions. It comprises questions on orientation, registration, short-term memory, language use, comprehension, and basic motor skills. The score ranges from 0–30. Patients are considered to be in mild stages of disease when scoring 20 points or above, to be in moderate stages of disease when scoring between 10 and 19, and severe when scoring 9 or less.

#### Clock drawing test [28]

The clock-drawing test is most commonly used to assesses the visual-spatial construction; however, successful completion of the task also requires verbal understanding, memory, and abstract reasoning. The patient is encouraged to draw a clock-face with clock-hands indicating the time “ten past eleven”. The clock drawn by a patient falls into one of the six categories, where categories 1 and 2 indicate a normal result and categories 3 to 6 indicate pathology.

#### Education

Educational attainment was categorized in basic, intermediate, and advanced education with 1 for education up to nine years, 2 for education up to 12 years, and 3 for education of 13 and more years.

#### Digit and visual span (Wechsler Memory Scale Revised, WMS-R)

The Digit Span test comprises digit span forward and digit span backward. Forward digit span is a measure of verbal short-term memory, defined as a system allowing temporary storage of the information. In this task participants are asked to repeat a sequence of digits until either the maximum number of eight digits per sequence was reached or until two consecutive incorrectly repeated sequences of same length. Backward digit span represents a measure of verbal working memory. In addition to storing information it also requires ability to manipulate and reproduce the information in an altered form. It employs the same procedure as forward digit span, except this time the numbers need to be repeated in a reversed order. Visual span is a visual-spatial alternative to measure same basic abilities using a different modality. It was measured using a Corsi block tapping test forward and backward. One point is given for each correct answer with scores ranging from 0–12 except for the forward visual span with scores ranging from 0–14.

#### Trail Making Tests A & B (TMT-A & TMT-B)

The Trail-Making-Test assesses visual attention and mental flexibility, which is a measure of executive functioning, and requires an examinee to draw pencil lines in ascending order from 1 to 25 (TMT-A) and 25 encircled numbers and corresponding letters in an alternating order (TMT-B) that are randomly dispersed on a DIN-A-4 sheet. The instructions require working as fast as possible while maintaining maximum accuracy. These tests measure the time to completion of the task. As recommended in the manual, we terminated the task at 350 s.

#### Fluency tasks (Regensburg Verbal Fluency Test; RWT) [29]

RWT assesses semantic and phonemic verbal fluency. It is used as a measure of lexical knowledge and lexical access speed as well as test of executive control ability. An examinee is instructed to generate as many words as possible in one minute that belong to the category *animals* (semantic verbal fluency) as well as words starting with the letters “*P”* and “*S”* (phonemic fluency).

#### California Verbal Learning Test (CVLT) [30]

The CVLT is a verbal memory test, assessing variables such as immediate recall, free and cued recall after short delay, free and cued recall after long delay as well as recognition. Taken together these variables are a good representation of episodic verbal memory and are sensitive to a range of memory deficits, such as problems in memory consolidation or retrieval. A list of 16 words (four words of each category: fruit, clothing, drinks, tools) is read to the participant a total of five times. After each round the participant is encouraged to recall as many words as possible. Immediate recall is followed by a free and cued delayed recall after 5 and 20 min intervals respectively, and a Yes/No recognition task.

### Statistical analyses

All statistical data analyses were carried out using the statistics program SPSS (SPSS 25.0 for Windows, Armonk, NY, 2017). The normality of distribution was tested with the Kolmogorov-Smirnov Test. Since all parameters were not normally distributed, group comparisons were calculated using nonparametric tests, mostly Mann-Whitney-U-test.

## Results

### Healthy controls

Overall, performance of HC (Table 1) is in good harmony with performance of older persons reported in the literature [31].

### Patients with depressive disorder

The whole group of patients with depressive disorder (cf. Fig 1; DD-all) comprises both patients with first time or recurrent depressive episode, and both patients on treatment (psychotherapeutic and/or drug treatment) or without treatment, and both patients with and without assessment of biomarkers of AD. Overall, patients belonging to the DD-all group performed worse than HC in all domains (Table 1).

A subgroup of patients was assessed with biomarkers of AD. This subgroup was split further into two groups. One group (DD-BM-) comprised only those patients with biomarker findings excluding AD. To obtain this group we chose conservative criteria including only patients in whom both Abeta1,42 was above diagnostic threshold (> 550 pg/ml) and levels of total-tau-protein were below diagnostic threshold (< 300 pg/ml), or patients in whom ^18^F-FDG-PET or amyloid-PET were negative. The other group of patients with analysis of biomarkers comprised patients in whom at least one biomarker was compatible with AD (DD-BM+; Abeta1,42 ≤ 550 pg/ml, total-tau-protein above diagnostic threshold (≥ 300 pg/ml), or ^18^F-FDG-PET suggestive of AD, or amyloid-PET positive). Overall, mean performance of patients from DD-BM+ was not different from patients from DD-BM- (Table 2).

**Table 2:** Mean performance between patients with DD-BM+ and DD-BM-.

	DD-BM-				DD-BM+					
N	90				86					
male/female	40 / 50				31 / 55					
education basic / intermedium / high / missing	68 / 15 / 6 / 1				59 / 16 / 9 / 2					
	Median	Min	Max	N	Median	Min	Max	N	Mann-Whitney-U-Test DD-BM- vs. DD-BM+ asymptotic significance	z-value
**Age (mean/SD)**	73,0	54,0	89,0	90	76,0	60,0	90,0	86	0.069	1.819
**MMSE (mean/SD)**	27,0	18,0	30,0	87	28,0	18,0	30,0	85	0.048	1.976
**GDS (mean/SD)**	6,0	0,0	15,0	85	7.5	0,0	15,0	80	0.316	1.004
**Digit Span forward**	6,0	1,0	12,0	86	6,0	2,0	12,0	83	0.786	-0.271
**Digit Span backward**	4,0	0,0	12,0	86	4,0	1,0	9,0	83	0.795	0.260
**Block Span forward**	6,0	1,0	12,0	86	6,0	3,0	10,0	81	0.883	-0.148
**Block Span backward**	5,0	1,0	10,0	86	5,0	0,0	9,0	81	0.533	0.623
**CVLT1**	3,0	1,0	9,0	86	4,0	0,0	7,0	85	0.191	1.309
**CVLT2**	5,0	1,0	11,0	86	5,0	0,0	11,0	85	0.925	-0.094
**CVLT3**	6,0	3,0	14,0	85	6,0	2,0	12,0	84	0.471	0.721
**CVLT4**	7,0	2,0	14,0	84	7,0	2,0	13,0	84	0.708	0.374
**CVLT5**	7,0	2,0	16,0	84	8,0	1,0	13,0	84	0.863	0.172
**CVLT total recall**	28,0	12,0	61,0	84	30,0	10,0	50,0	84	0.645	0.460
**CVLT del. free recall**	4,0	0,0	15,0	84	4,0	0,0	14,0	83	0.908	-0.116
**CVLT del. cued recall**	6,0	1,0	16,0	84	6,0	1,0	16,0	83	0.990	-0.013
**CVLT recognition**	14,0	5,0	16,0	83	15,0	4,0	16,0	82	0.211	1.250
**CVLT false positve**	2,0	0,0	19,0	83	2,0	0,0	21,0	81	0.652	-0.451
**Clock drawing**	3,0	1,0	5,0	87	3,0	1,0	5,0	82	0.846	-0.194
**TMT-A**	61.5	26,0	180,0	86	75,0	28,0	262,0	82	0.077	1.769
**TMT-B**	163,0	52,0	325,0	77	190.5	83,0	300,0	64	0.166	1.387
**Semantic Fluency**	14,0	5,0	30,0	85	15,0	5,0	28,0	82	0.457	0.744
**Phonematic Fluency P**	6,0	0,0	19,0	85	6,0	0,0	15,0	82	0.676	0.418
**Phonematic Fluency S**	7,0	0,0	20,0	83	8,0	1,0	23,0	80	0.461	0.738

CVLT: California Verbal Learning Test; GDS: 15-item Geriatric Depression Scale; MMSE: Mini-Mental-Status-Examination; Semantic fluency: category animals; TMT-A and TMT-B: Trail-Making-Test A and B

Biomarker assessments for DD-BM- and DD-BM+ are shown in Table 3.

**Table 3:** Biomarker assessments for DD-BM- and DD-BM+.

	DD-BM-				DD-BM+					
n	90				86					
male/female	40 / 50				31 / 55					
education basic / intermedium / high / missing	68 / 15 / 6 / 1				59 / 16 / 9 / 2					
	Median	Min	Max	N	Median	Min	Max	N	Mann-Whitney-U-Test DDBM- vs. DDBM+ asymptotic significance	z-value
**CSF**										
**Abeta 1,42**	834.5	579	1580	56	716	224	1815	83	0.013	-2.497
**Abeta 1, 40**	6900	3150	10800	43	6740	1930	19666	59	0.924	-0.095
**Amyloid quotient**	0.12	0.08	0.19	44	0.11	0.05	0.15	62	0.000	-3.694
**total tau-protein**	231	81	300	56	359	15	1290	84	0.000	6.714
**phospho-tau-protein**	42	21	68	29	54	19	94	47	0.104	1.626
**18F-FDG-PET**				33 / 0 +				8 / 2 +		
**Amyloid-PET**				11 / 0 +				6 / 0 +		

DD-BM-: Abeta1,42 > 550 pg/ml AND total-tau-protein < 300 pg/ml or 18F-FDG-PET negative or Amyloid-PET negative; DD-BM+: at least one biomarker (Abeta1,42, total tau-protein, phospho tau-protein, 18F-FDG-PET, or Amyloid-PET positive. Amyloid quotient: level of Abeta1,42 / level of Abeta1,40.

### Matched cohorts of patients with depressive disorder and negative biomarkers and whole group of healthy controls

Lastly, we matched groups of HC and patients with DD-BM- for age (± 3 years), education (± 1), MMSE (± 1), and biological gender (exact). Matching was performed with a 1:1 match. Even in this group, performance of patients with DD was below that of HC (Table 4).

**Table 4:** Matched groups of HC and patients with DD-BM-.

	HC				DD-BM-					
n	40				40					
male/female	21 / 19				21 / 19					
education basic/intermedium/high	16 / 15 / 9				25 / 10 / 5					
	Median	Min	Max	N	Median	Min	Max	N	Mann-Whitney-U-Test HC vs. DD-BM- asymtotic significance	z-value
**Age (mean/SD)**	69,0	52,0	80,0	40	68,0	54,0	81,0	40	0.675	0.419
										
**MMSE (mean/SD)**	30,0	28,0	30,0	40	28,0	21,0	30,0	39	0.000	-5.833
**GDS (mean/SD)**	2,0	0,0	8,0	38	8,0	1,0	15,0	38	0.000	5.885
**Digit Span forward**	8,0	4,0	12,0	40	7,0	1,0	12,0	38	0.051	-1.951
**Digit Span backward**	6,0	2,0	11,0	40	4,0	1,0	12,0	38	0.005	-2.780
**Block Span forward**	7,0	4,0	10,0	40	6.5	1,0	10,0	38	0.594	-0.533
**Block Span backward**	6.5	4,0	9,0	40	4,0	2,0	10,0	38	0.000	-3.921
**CVLT1**	5,0	2,0	9,0	40	3,0	1,0	6,0	38	0.000	-3.996
**CVLT2**	8,0	4,0	12,0	40	5,0	2,0	10,0	38	0.000	-4.124
**CVLT3**	10,0	4,0	14,0	40	5.5	3,0	14,0	38	0.000	-4.792
**CVLT4**	10.5	4,0	16,0	40	7,0	3,0	13,0	38	0.000	-4.852
**CVLT5**	12.5	1,0	16,0	40	8,0	2,0	16,0	38	0.000	-4.753
**CVLT total recall**	45.5	24,0	64,0	40	28.5	12,0	56,0	38	0.000	-5.180
**CVLT del. free recall**	11,0	2,0	16,0	40	5.5	0,0	13,0	38	0.000	-5.059
**CVLT del. cued recall**	12.5	6,0	16,0	40	7.5	3,0	16,0	38	0.000	-4.862
**CVLT recognition**	16,0	13,0	16,0	40	14,0	9,0	16,0	38	0.000	-3.807
**CVLT false positve**	0,0	0,0	2,0	40	2,0	0,0	18,0	38	0.000	4.430
**Clock drawing**	1,0	1,0	4,0	40	2,0	1,0	5,0	38	0.000	3.899
**TMT-A**	39.5	21,0	139,0	40	49,0	26,0	180,0	39	0.014	2.453
**TMT-B**	97,0	28,0	270,0	39	134,0	52,0	300,0	33	0.003	3.018
**Semantic Fluency**	20.5	12,0	31,0	40	16,0	5,0	30,0	39	0.002	-3.058
**Phonematic Fluency P**	9,0	1,0	18,0	40	7,0	0,0	19,0	39	0.004	-2.884
**Phonematic Fluency S**	12,0	2,0	21,0	37	8,0	3,0	20,0	37	0.003	-2.927

CVLT: California Verbal Learning Test; GDS: 15-item Geriatric Depression Scale; MMSE: Mini-Mental-Status-Examination; Semantic fluency: category animals; TMT-A and TMT-B: Trail-Making-Test A and B; DD-BM-: Abeta1,42 > 550 pg/ml AND total-tau-protein < 300 pg/ml or 18F-FDG-PET negative or Amyloid-PET negative; HC: Healthy controls

With a criterion of performance being below mean minus 1.5 standard deviations indicating pathology [32] a percentage of 50.0% and 57.9% of patients with DD were below normal for total and delayed recall in the CVLT. With the same criterion 30.3%, 35.9%, and 28,2% of patients were below normal in the TMT-B, semantic fluency (category animals), and phonemic fluency (letter “P”), respectively.

## Discussion

To our knowledge, this is the first study to characterize the pattern of cognitive impairment in patients with depressive disorder (DD) in whom the most frequent cause of cognitive impairment in older age, AD, has been ruled out.

Subjective memory complaints have been found useful to detect early AD [33]. Likewise objective screening tests have been found useful [34]. In fact it seems reasonable to not rely on subjective memory impairment to decide on whom further procedures are initiated but rather to include the results of objective tests in this decision [35]. The latter procedure resembles that taken by the present study with taking the subjective complaints seriously, performing elaborate neuropsychological testing, and then deciding on further procedures.

History-taking in older persons with DD is difficult and duration of previous depressive episodes and age at onset of depressive disorder cannot always be determined reliably even when informants and structured interviews are used [36]. The literature on the impact of prior depressive episodes and early-onset vs. late-onset depressive disorder on cognitive performance in older persons with DD is ambiguous. It was reported that late-onset disorder is associated with worse executive functioning [37] but it was also reported that the late-onset group differs in poorer performance on measures of verbal learning and memory with executive functioning being similar to the early-onset group [38]. Yet another study reported that a history of past depression was associated with worse executive function but that controlling for psychological distress diminished this association [39]. The focus of the present study was to characterize the cognitive profile in patients with DD in whom the most frequent cause of cognitive impairment in older age, Alzheimer’s disease, was ruled out. A priori exclusion of patients with unknown numbers of episodes or comorbid conditions would not represent the majority of patients and thus would not be ecologically valid. The present study lays ground for a prospective study addressing the impact of prior depressive episodes on cognitive function in persons with ‘pure’ DD.

We assessed cognitive performance of three groups of patients with DD. The group DD-all comprises the whole group of patients with the diagnosis of DD regardless of whether biomarker analysis was performed or not. The group DD-BM+ describes a group of patients with DD in whom at least one biomarker was positive (CSF biomarkers (Abeta1,42, amyloid ratio (level of Abeta1,42 / level of Abeta1,40), levels of total tau- and phospho-tau-protein), FDG-PET, and amyloid-PET). The group DD-BM- includes only patients with DD in whom all biomarkers were within normal range. Possible AD may be diagnosed when clinical symptoms are consistent with AD and at least one CSF biomarker is positive with others unknown; probable AD may be diagnosed when clinical symptoms are consistent with AD and two CSF biomarkers or imaging biomarkers are positive [40]. We chose this approach because it was our interest to analyze patients with DD in whom other causes of depression, such as neurodegenerative dementia, were excluded at its best.

Surprisingly, the group of patients with biomarkers within normal range (DD-BM-) and the group with at least one positive biomarker (DD-BM+) were alike in all cognitive domains (Table 2). This finding likely reflects the low informative value of an isolated positive biomarker finding. Therefore, the group of patients with one positive biomarker is indeterminant and heterogenous. It comprises both patients with DD in whom the biomarker is an auxiliary finding not sufficient to justify a diagnosis other than DD and patients in whom a possible neurodegenerative process as indicated by the positive biomarker is too mild to affect cognitive performance.

In order to further analyze the cognitive performance of patients with pure depression (DD-BM-) we matched this group with HC. With a criterion of mean minus 1.5 standard deviations and below of HC values indicating impairment [32], we found about one third of subjects with DD to be impaired in executive function and about two out of three subjects to be impaired in total recall and delayed recall.

Previously it was argued that the pattern of cognitive deficits in patients with DD is most pronounced for recall tasks but largely intact for consolidation of information over a delay period [41]. The present study, however, demonstrates a pronounced impairment of memory consolidation as well as of recall. Possibly, previous studies investigating the neuropsychological pattern of patients with DD excluded patients with deficits in memory consolidation as being suggestive of AD without securing the diagnosis of AD by performing CSF analysis.

There are some limitations to the present study. The value of using the MMSE to screen for cognitive impairment in patients with DD is questionable [42]. However, the procedure in the present study was to use the MMSE as a criterion as to whether a patient should be subjected to detailed neuropsychological testing. With a MMSE score below 22 comprehensive neuropsychological testing cannot be performed due to a lack of ability to comply with test instructions and reaching floor performance on testing. Being a retrospective cohort study the DD group comprises both treated and untreated patients. Our medical records did not allow us to obtain sufficient information on drug treatment at the time of neuropsychological testing to address the extent to which drug treatment might have had an impact on our results. In older patients several classes of drugs are discussed to impact cognitive performance (e.g. antidepressants, antipsychotics, benzodiazepines, beta-blockers, urological and other anticholinergic drugs). Our records do not allow us to reconstruct all drugs, dosage, and time interval to last drug administration prior to neuropsychological examination. Although some studies have reported that short-term use of benzodiazepines in patients with depressive disorder does not impair cognitive function [43, 44] and low dose antipsychotics do not impair cognitive function in patients with cognitive impairment [45] we consider the lack of data on drugs a limitation that needs to be addressed in a future prospective study. Another limitation of the present study is the lack of information on the number of depressive episodes in our routine medical records because it has been reported that the severity of cognitive impairment increases with number of depressive episodes in patients with depressive disorder [46]. Furthermore, no pre-specified algorithm was used to decide on whom to perform a CSF tap or assessment with ^18^F-FDG-PET or amyloid-PET other than the criterion of medical history being suggestive of cognitive impairment and neuropsychological testing objectifying the impairment. On the other hand, this lack of a precise algorithm reflects the vague knowledge about profiles of cognitive impairment due to DD or due to AD in older patients. The similarity of the cognitive profile in the groups DD-all and DD-BM- support the quality of the clinical diagnosis of DD in our sample.

The hallmark of deficits in AD is an impairment in total recall and delayed recall [47]. The present study demonstrates that these capabilities are also the areas most impaired in cognitive impairment due to DD. Although the CVLT is considered to be the most sensitive neuropsychological marker to differentiate normal aging from cognitive impairment [48], the present study demonstrates that determining the etiological cause of memory deficits is not possible on neuropsychological grounds. Diagnosis of cognitive impairment in older persons requires analysis of neuropsychological as well as of biological parameters. Thus, the clinical algorithm for older subjects with DD and cognitive symptoms should comprise a CSF tap or other biomarker assessments in order to rule out or secure diagnosis of AD.

## Conclusion

In summary, the present study is the first to demonstrate cognitive impairment due to DD in a large sample of older patients after excluding patients with comorbid AD. The cognitive profile in older patients with DD without and with biomarkers of AD is not distinguishable. Therefore, cognitive impairment due to DD should be diagnosed only after exclusion of AD biomarkers. Cognitive impairment due to DD should receive more attention in psychiatric algorithms and diagnostic criteria.

## Supporting information

S1 Data (XLSX)

S1 File (DOCX)

## Abbreviation list

Am-PET: Amyloid-PET
CVLT: California Verbal Learning Test
DD: Depressive Disorder
DD-all: all patients with depressive disorder
DD-BM: patients with depressive disorder with no pathognomonic biomarker of AD
DD-BM+: patients with depressive disorder with at least one biomarker indicating AD
FDG-PET: ^18^F-Fluordesoxyglucose positron emission tomography
GDS: Geriatric Depression Scale
HC: Healthy old controls
MMSE: Mini-Mental Status Examination
