# Supplementary material for: On the conundrum of cognitive impairment due to depressive disorder in older patients
Source: PLoS One. 2020 Apr 2;15(4):e0231111. doi: 10.1371/journal.pone.0231111 (PMC7117703; doi:10.1371/journal.pone.0231111)
Supplement: S1 File — (DOCX) [file pone.0231111.s002.docx]

Since numbers in Figure 1 are only descriptive in nature, no statistical analysis was performed.
